# Molecular description of non-autoimmune hyperthyroidism at a neonate caused by a new thyrotropin receptor germline mutation

**DOI:** 10.1186/1756-6614-4-S1-S8

**Published:** 2011-08-03

**Authors:** Heike Biebermann, Franziska Winkler, Daniela Handke, Annette Grüters, Heiko Krude, Gunnar Kleinau

**Affiliations:** 1 Institute of Experimental Paediatric Endocrinology, Charité Universitätsmedizin Berlin, Augustenburger Platz 1, 13353 Berlin, Germany

## Abstract

**Background:**

Constitutively activating germline mutations in the thyrotropin receptor (TSHR) gene result in non-autoimmune hyperthyroidism and can be transmitted as a dominant trait or occur sporadically. These mutations are mostly located in the serpentine part of this G-protein coupled receptor.

**Methods:**

Sequencing exon 9 and 10 of the thyrotropin receptor gene in a two months old patient identified a mutation which was functionally characterized after transient transfection into COS-7 cells. Cell surface localization was investigated by an ELISA approach and for signalling properties we measured cAMP by alpha screen technology for Gs/adenylyl cyclase activation and use a reporter gene assay for determination of Gq/11 phospholipase C-β activation.

**Results:**

We detected a heterozygous mutation in the first extracellular loop of the TSHR gene leading to an exchange of an isoleucine residue for asparagine at amino acid position 486 (I486N). Cell surface localization was reduced to 51% of wild-type TSHR. Functional characterization of the mutant receptor revealed constitutive activation of the Gs/adenylyl cyclase pathway, in contrast basal activity of the Gq/11 pathway was comparable to the wild-type. The bovine TSH-induced cAMP accumulation was slightly reduced, but IP3 signaling was impaired.

**Conclusion:**

We identified a new TSHR germline mutation (I486N) in a neonate with non-autoimmune sporadic hyperthyroidism. The mutation is located at the extracellular loop 1 and exhibits an increase in basal cAMP accumulation, but unexpectedly impairs the capability for TSH induced Gq mediated signaling. The TSHR homology model suggests isoleucine 486 as a potential key-player for induction of signal transduction by an interplay with further activation sensitive extracellular parts.

## Introduction

The thyrotropin receptor (TSHR) is a member of the glycoprotein hormone receptors (GPHRs), a subfamily of the family A G-protein-coupled receptors (GPCRs). In contrast to most other receptors of this family the GPHRs are characterized by a large amino-terminal extracellular part of around 350 amino acids containing high affinity binding sites for hormones and antibodies. The amino-terminal extracellular part can be subdivided into the so-called Leucine-rich repeat domain and the hinge region. The serpentine domain is constituted by seven transmembrane α-helices (TMHs) connected by three intra- (ICLs) and three extracellular (ECLs) loops (reviewed in [1]). The intracellular serpendine domain together with the intracellular C-terminus are involved in G-protein activation.

Hormone binding on the amino-terminal extracellular part induces conformational changes within the 7TMH leading to an active conformation and signaling-state of the receptor [2]. The TSHR couples mainly Gαs to induce activation of adenylyl cyclase, but at higher TSH concentrations also activates the inositol phosphate cascade (IP) via coupling to Gαq [3,4]. So far activation of the Gs/adenylyl cyclase pathway seems to be of highest impact for thyroid growth and function [5]. However, recently it was shown that activation of the Gq/11 pathway is likely an important factor for thyroid growth and thyroid hormone synthesis [6].

Constitutively activating mutations (CAMs) of the TSHR result in non-autoimmune hyperthyroidism. These mutations are leading to a permanently increased stimulation of the Gs/adenylyl cyclase pathway. The majority of germline CAMs are located in the serpendine domain, but few of such mutations were also found in the ECLs (reviewed in [7]). When occurring in the germline these mutations are found as sporadic carriers or are inherrited as dominant traits in familial cases. Somatic mutations in hyperfunctioning nodules are extremely rare in pediatric patients [8,9]. The phenotypic variability of mutations carriers is ranging from slight signs of hyperthyroidism in later life (mostly in familial occurring mutations) to severe forms of hyperthyroidism in neonates [8].

Here we describe a neonate in whom enhanced tachycardia was the only clinical sign of hyperthyroidism, which is due to the lack of TSH-R stimulating antibodies and was diagnosed as non-autoimmune hyperthyroidism. A new constitutively activating germline TSHR mutation, localized in the ECL1 was identified. Here we describe the patient`s-phenotype, results of molecular characterization and modified structural-functional properties.

## Material and methods

### The patient

The patient was born after an uneventful pregnancy in the 36 week of gestation without signs of prior fetal tachycardia. In utero ultrasound data of the thyroid gland were not available. At the age of one month tachycardia was detected in a routine examination without further clinical signs of hyperthyroidism. At one month of age the weight was 3085 g and length 52 cm length. The thyroid status revealed severely elevated T3 (5.5 µg/L; normal range: 0.8 – 2.0 µg/L) and fT4 (≥ 7.8 ng/dL; normal range: 0.9 – 1.9 ng/d) levels, and supposed TSH below 0.01 mU/L (normal range: 0.27 – 4.2 mU/L). Thyroid autoantibodies were negative. Ultrasound of the thyroid gland showed no increase in volume (1.9 mL; normal range: 0.2 – 2.0 mL).

Due to severe tachycardia with 220/min the child was treated with Propanolol 3.1 mg/kg body weight/d. Antithyroid drug treatment with Thiamazol was initiated with a dose of 0.5 mg/kg body weight per day. In light of severe hyperthyroidism in terms of very high thyroid hormone levels the clinical course beside severe tachycardia was surprisingly asymptomatic including the relatively small volume of the thyroid gland. Actually at the age of two months the patient was treated with 2.5 mg Propanolol four times a day, and with Thiamazol 1 mg/d and D-Fluorette 500 IE/d.

All clinical investigations described were conducted in consultation with the investigated patient and her family and genetic analyses were in accordance with the guide-lines proposed in the Declaration of Helsinki.

### Isolation and characterization of genomic DNA encoding the TSHR

Genomic DNA was prepared from peripheral white blood cells from the patient using a commercial kit (Blood amp kit, Qiagen, Hilden, Germany). Exon 9 and 10 of the TSHR gene were PCR-amplified and sequenced using the BigDye Terminator Cycle Sequencing Ready Reaction Kit (Perkin Elmer, Weiterstadt, Germany) and an automatic sequencer (ABI 3710xl, Applied Biosystems, Foster City, CA, USA).

### Cloning of TSHR mutation

The PCR product of the mutation I486N was cloned into the pCR2.1-TOPO vector and then transformed into competent *E*.*coli* DH5α. DNA was extracted from selected colonies of bacteria using QIAprep Miniprep kit (QIAGEN GmbH, Germany), digested with *Eco*RI (New England Biolabs, MA, USA) and additionally sequenced for the detection of mutation I486N. The mutant was inserted into the eucaryotic expression vector encoding the wild-type (WT) TSHR (N-terminal hemagglutinine (HA)-tagged between amino acid 25 and 26) via Bsu36I and BstEII.

### Cell culture, transfection and functional assays

COS-7 cells were grown in Dulbecco´s modified Eagle medium (DMEM) and HEK-293 cells in Minimum Essential Medium (MEM) Earle´s, both supplemented with 10% fetal bovine serum (FBS), 100 U/ml penicillin, 100 µg/ml streptomycin and 2 mM L-glutamine at 37°C in a 5% CO_2_ incubator. For functional characterization WT TSHR and mutant receptor were transiently transfected into COS-7 or HEK 293 cells and investigated regarding cell surface expression and TSH-induced accumulation of intracellular cAMP and IP3 accumulation.

For all functional tests 2.5x10^4^ COS-7 cells (for cAMP accumulation) or 5 X 10^4^ HEK 293 cells (for IP accumulation) were seeded in 48-well culture plates. Transient transfection was performed by using metafectene (Biontex, Munich, Germany) according to the manufactures protocol. Cell surface expression was determined by immunological detection of the N-terminal HA-tag as described previously [10]. Intracellular cAMP was determined via alpha-screen technology [11] after stimulation with bovine TSH (bTSH). For investigation of Gq/11 activation, we co-transfected HEK-293 cells with TSHR cDNAs and a reporter construct containing the firefly luciferase gene under the control of NF-AT. Subsequently, the cells were stimulated with bTSH and lysed. Gq/11 activation was reported by luciferase activity expression in the luciferase reporter gene assay according to the manufacturer’s instructions (Promega, Madison, USA).

### Design of a molecular TSHR homology model

Using a previously described modeling procedure [12] we designed a new TSHR homology model of the serpentine-domain assembled with the extreme N- and C-terminal parts of the hinge region. As suggested by recently published experimental data [13] a cysteine-bridge between C284 at the N- and C408 at the C- terminus of the hinge region was added. To improve the model and to increase the quality (refined side-chain orientations) we performed in contrast to the previous procedure an extended molecular dynamic simulation of 6ns for the side-chains by fixed backbone atoms. Structure images were produced using the PyMOL Molecular Graphics System, Version 1.3, Schrödinger, LLC.

## Results

### Sequencing of the TSHR gene

Direct sequencing of exon 10 of the TSHR gene in the patient revealed a heterozygous mutation, c.T1458A, that resulted in an exchange of p.**Ile486Asn** in the first extracellular loop. This mutation was not identified in the parents.

### Functional characterization of wild type and mutant TSHR (I486N)

For functional characterization the mutated TSHR (I486N) and wild type TSHR were transiently transfected into either COS-7 cells to detect cell surface localization and cAMP signal transduction or HEK-293 cells to investigate IP3 signaling properties.

### Cell surface localization

**Figure 1:**
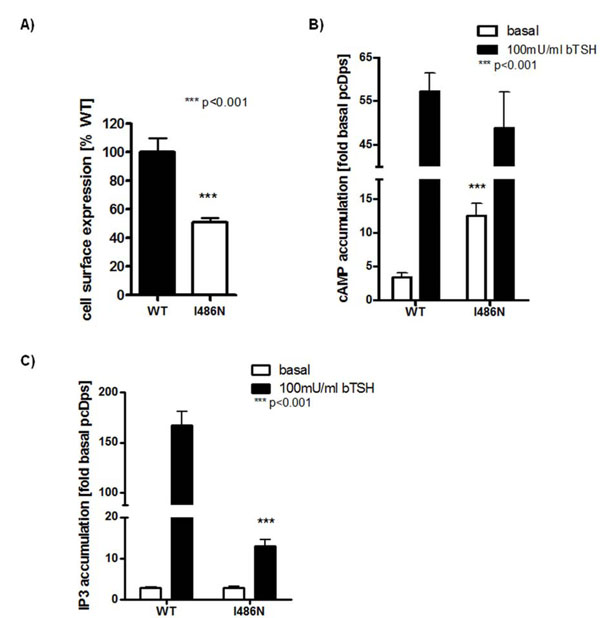
Functional characterization of WT and mutant TSHR. Cell surface expression (A) and Gs cAMP accumulation (B) were determined in COS-7 cells; Gq/_11_ IP3 accumulation (C) was determined in HEK-293 cells, both expressing WT and mutant (I486N) TSHRs. The cells were transiently transfected with WT TSHR and mutant TSHR-I486N DNA. A: Cell surface expression was measured 72 h after transfection. The cells were fixed, and immunological determination of the N-terminal HA was performed. Results (means ± SD) are expressed as percentage of TSHR-WT (100%). B: Activation of the Gs/adenylyl cyclase pathway was determined 48 h after transfection. Cells were stimulated with bTSH, and intracellular cAMP accumulation was measured. Basal activity is indicated in white bars; activity after stimulation with 100 mU bTSH/ml is indicated in black bars. C: Gq/11 activation was reported 48 h after COS-7 co-transfection with TSHR DNA and a reporter construct containing a response element and the firefly luciferase gene under the control of nuclear factor of activated T-cells (transcription factor). The cells were stimulated with bTSH and lysed, and luciferase activity expression was measured. Basal activity is indicated in white bars, activity after stimulation with 100 mU bTSH/ml in black bars. The figure depicts means ± SD out of duplex or tripletts (n= 2-3). RLU, Relative light units. (*** means p value <0.001). The statistical analysis was made by using a nonparametric One-way-Anova and a tukey-test for column statistic of GraphPad Prism Version 4.03.

The mutant TSHR expression at the cell surface was reduced to 51% ± 7% of WT TSHR (Fig.1 A).

### Signaling properties

We determined a 4-fold increase in ligand independent activation of the Gαs signaling pathway compared with the WT (I486N: 404% ± 67% of WT; Fig.1B). The maximal level of activation of the mutant was slightly reduced compared with WT (I486N: 74% ± 8% of WT; Fig. 1B), whereby the EC_50_ values were similar (EC50 WT: 0.18 ± 0.02 mU/ml; I486N: 0.35 ± 0.17 mU/ml).

In contrast, determination of basal and TSH- induced activation of the Gαq/11 signaling pathway revealed a 13-fold impairment of signaling capability of the I486N mutation (I486N: 8% ± 2% of WT). The basal activity was like wild type (Fig.1C).

### The TSHR homology model suggests an interplay between isoleucine 486 at the extracellular loop 1 with fragments of the extracellular hinge region

**Figure 2:**
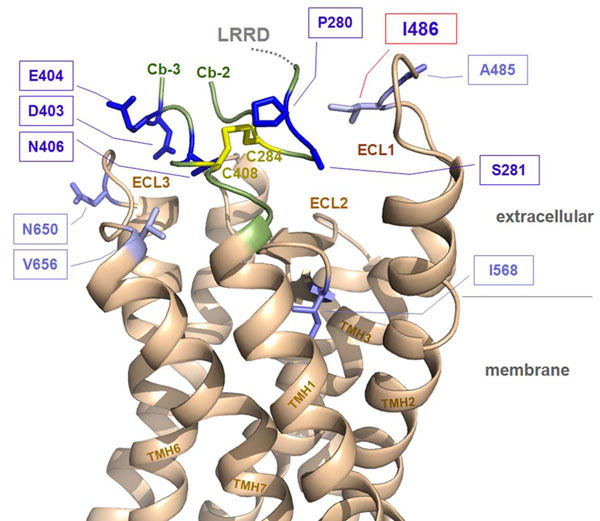
This TSHR homology model (backbone-ribbon presentation) visualizes the potential arrangement of different structural parts at the interface between the extracellular and transmembrane region. Naturally occurring activating mutations in the extracellular loops (ECLs) were reported several times for the TSHR: ECL1- at positions I486 (red boxed) and A485; ECL2 - at position I568; ECL3 at positions N650 and V656 (wild type amino acids are shown as light blue sticks). Interestingly, the wild type amino acids side-chains are exclusively hydrophobic. The tightly embedded structural-functional counterparts of the loops are the extreme N- and C-terminus (green backbone) of the extracellular hinge region (cysteine-boxes 2 and 3 (Cb-2, Cb-3)) were also activating mutations are known (wild type residues as blue sticks). In conclusion these receptor components are sensitive for activation and they are assembled tightly together at the extracellular site of the receptor. Activation of the TSHR on the extracellular site might be related to a relative spatial shift of these determinants to each other, which mediates signal-transduction to the transmembrane helices.

It can be assumed that the extreme N- and C-terminus of the extracellular hinge region are localized and tightly embedded between the ECLs (figure 2). Three facts do support this three-dimensional package:

1. The C-terminus of the hinge region directly attaches the extracellular part to the transmembrane helix 1 (TMH1).

2. The experimentally evidenced cysteine bridge (C284-C408) connects the C- with the N-terminus of the hinge region.

3. It has been shown [14,15] that the TSHR without the AtEP can not become activated by mutations at the extracellular loops. At the ECLs as well as on the N- and C-terminus of the hinge region activating mutations were found, which supports there potential interplay as counterparts at the interface between the extracellular and transmembrane region during TSHR activation.

## Discussion

### Occurrence of activating mutations in the first extracellular loop of the Thyrotropin receptor

Constitutively activating mutations in the TSHR are a frequent molecular cause of non-autoimmune hyperthyroidism (reviewed in [16-19]). We here describe a new germline TSHR mutation I486N located in the ECL1. In the affected neonate hyperthyroidism was diagnosed due to tachycardia without further signs of hyperthyroidism and without goiter. Thyroid hormone values were severely elevated revealed non-autoimmune hyperthyroidism.

Three somatic mutations at position 486 were found previously in toxic thyroid adenomas: I486F [20], I486M [20], I486N [21]. Functional characterization for mutations I486F and I486M demonstrated constitutive activation of both Gs and Gq mediated signaling. The mutation I486N we identified in this study as a germline mutation was already found as a somatic mutation [21]. In that study mutation I486N lead to constitutive activation of the Gs pathway, however, activation of Gq activation was not investigated. We here show that I486N did not result in constitutive activation of Gq/11 (figure 1) in contrast to the I486F and I486M mutations. Moreover, the capability for bTSH-induced Gq-mediated signaling was impaired in the I486N mutation.

Interestingly, at the neighbouring position 485 a germline mutation (A485V) was described previously [22]. This mutation also reveals constitutive activation of the Gs mediated pathway, whereas the basal level of IP3 formation remained unchanged. Stimulation of that mutant TSHR with high concentrations of bTSH failed to activate the IP3-Gq pathway. The patient presented with elevated thyroid hormone levels and a suppressed TSH level in the absence of thyroid hormone antibodies, leading to the diagnosis of non-autoimmune hyperthyroidism. No measurable goiter was found in this patient and the phenotype was described as a mild form of hyperthyroidism, although the functional characterization revealed a high constitutive basal activity. This phenotype and functional characterization of the mutated TSHR is similar to our findings regarding the patient-phenotype and molecular characteristics of the TSHR variant I486N. We recently described a patient`s phenotype due to a TSHR mutation with comparable functional characteristics as non-autoimmune and non goitrous hyperthyroidism [23].

### Implications from naturally occurring mutations for structural-functional aspects of the Thyrotropin receptor and patient phenotypes

To understand the functional and structural role of TSHR-I486N consideration of naturally occurring TSHR mutations in the extracellular loops provides valuable information. Naturally occurring activating mutations in the ECLs are reported several times in the TSHR: ECL1- I486M,F,N [20,21] and A485V [22]; ECL2 - I568T,V [20,24], ECL3 - N650Y [25] and V656F [26]. These activating mutations are indicators that the extracellular loops acting as important determinants for receptor activation. In support to this assumption also few naturally occurring inactivating mutations were published previously: ECL1 - W488R [27], Q489H [28]; ECL3 - L653V [29].

The potential interplaying counterparts of the ECLs (figure 2) are likely the extreme N- and C-terminus of the extracellular hinge region where also activating as well as inactivating mutations were reported (reviewed in [1]). Mutations at I486 likely modify the conformation of loop 1 and by this lead to re-justification of the extracellular receptor parts to each other. This spatial shift induces interruption of side-chain interactions or induces new interactions between amino acids which finally effects the arrangement of connected transmembrane helices (intramolecular signal transduction). The specific importance of the TSHR ECL1 in this scenario was evidenced and specified by site-directed mutagenesis studies [15,30]. In these studies the ECL1 was identified as a key-transducer and amplifier of extracellularly provided signals, which is supported by findings at other GPCRs [31,32].

Of note are the diverse functional characteristics of different I486 mutations tested in cell-systems. Two observations which might be exemplarily also for other pathogenic mutations we would like to highlight:

1. The here described mutation I486N on one hand constitutively activates the Gs pathway, but on the other hand selectively impairs the capacity for bTSH to induced Gq activation. Such selective inactivation for the hormone induced Gq mediated pathway is observed also for the inactivating pathogenic mutation L653V at the ECL3 [29]. Finally, this implicates that on one hand activating, on the other hand inactivating properties for a single *s*ide-chain substitution has to be considered. Such dual signal modification is reported recently also for the transmembrane mutant C636W [33]. Unfortunately in most cases of naturally occurring mutants a comprehensive experimental characterization is still missing (see information resource at http://www.ssfa-gphr.de).

2. Furthermore, functional comparison of different mutations at TSHR position 486 reveals substitution-dependent signaling properties. In opposite to mutations I486M and I486F the I486N mutation does not induce constitutive IP accumulation and simultaneously reduces the capacity for Gq activation after TSH binding. This contrast is likely related to the different biophysical properties of the particular substitutions. The methionine and phenylalanine residues are different in bulge and side-chain flexibility, but hydrophobic compared with the wild type isoleucine. In contrast, the hydrophilic asparagine can establish H-bonds or causes repulsion in a hydrophobic micro-environment. In consequence different effects on signaling compared to Met or Phe mutants are to be observed. Strikingly, these findings indicating a position and region were this receptor can be modified selectively for specific signaling properties and this might have impact also on the patient`s thyroid function and thereby the patient`s phenotype.

## Conclusion

Reported activating somatic or germline mutations in the extracellular loops show variable forms of hyperthyroidism-phenotypes. Even the phenotypical variability among carriers of the same germline mutation or even among mutation carriers of one family [22,34] could range from onset of hyperthyroidism as neonate and slight sign of hyperthyroidism as adult [34]. Potential explanations may result from above described differences in the modification of signaling pathways [35], but definitively there is no direct link between the degree of constitutive cAMP activation caused by a certain mutation obtained in an artificial *in vitro* over-expressing system and the severity of hyperthyroidism in patients [26,36]. Therefore it might be that estimation of further molecular aspects of TSHR signal transduction should help to explain the variability in hyperthyroidism-phenotypes.

## Abbreviations

bTSH: bovine TSH; cAMP: cyclic adenosine monophosphate; CAM: Constitutively activating mutations; DMEM: Dulbecco´s modified Eagle medium; ECL: extracellular loop; FBS: fetal bovine serum; fT4: free T_4_; GPCR: G protein-coupled receptor; GPHR: glycoprotein hormone receptor; HA: hemagglutinin; ICL: intracellular loop; IP: inositol phosphate; IP3: inositol trisphosphate; MEM: Minimum Essential Medium Earle´s; NF-AT: nuclear factor of activated T-cells; TMH: transmembrane helix; TSH: thyroid-stimulating hormone; TSHR: TSH receptor; WT: wild-type.

## Competing interests

The authors confirm that there are no competing interests.

## Authors’ contributions

HB and GK designed and coordinated the study and qualified the patients together with HK and AG. FW and DH did the functional characterization of the TSHR (WT and mutant) and performed statistical analysis. GK designed the TSHR homology model. All authors wrote the manuscript. All authors read and approved the final manuscript.
